# In vitro osteogenesis of rat bone marrow mesenchymal cells on PEEK disks with heat-fixed apatite by CO_2_ laser bonding

**DOI:** 10.1186/s12891-020-03716-1

**Published:** 2020-10-19

**Authors:** Sachiko Kawasaki, Yusuke Inagaki, Manabu Akahane, Akira Furukawa, Hideki Shigematsu, Yasuhito Tanaka

**Affiliations:** 1 Department of Orthopaedic Surgery, Nara Medical University, Shijocho 840, Kashihara, Nara 634-8522 Japan; 2 Department of Health and Welfare Services, National Institute of Public Health, South 2-3-6, Wako, Saitama, 351-0197 Japan

**Keywords:** Polyether-ether-ketone, CO_2_ laser bonding, Osteogenesis, Apatite

## Abstract

**Background:**

Polyether-ether-ketone (PEEK) is increasingly being used for spinal applications. However, because of its biologically inactive nature, there are risks of false joint loosening and sinking. PEEK materials are coated with apatite to enhance the osteoconductive properties. In this study, we aimed to evaluate whether strontium apatite stimulate osteogenesis on the surface of PEEK by using the CO_2_ laser technique.

**Methods:**

We prepared non-coated disks, laser-exposed disks without apatite, and four types of apatite-coated by laser PEEK disks (hydroxyapatite (HAP), strontium hydroxyapatite (SrHAP), silicate-substituted strontium apatite (SrSiP), and silicate-zinc-substituted strontium apatite (SrZnSiP)). A part of the study objective was testing various types of apatite coatings. Bone marrow mesenchymal cells (BMSCs) of rats were seeded at a density of 2 × 10^4^/cm^2^ onto each apatite-coated, non-coated, and laser-irradiated PEEK disks. The disks were then placed in osteogenic medium, and alkaline phosphatase (ALP) staining and Alizarin red staining of BMSCs grown on PEEK disks were performed after 14 days of culture. The concentrations of osteocalcin (OC) and calcium in the culture medium were measured on days 8 and 14 of cell culture. Furthermore, mRNA expression of osteocalcin, ALP, runt-related transcription factor 2 (Runx2), collagen type 1a1 (Col1a1), and collagen type 4a1 (Col4a1) was evaluated by qPCR.

**Results:**

The staining for ALP and Alizarin red S was more strongly positive on the apatite-coated PEEK disks compared to that on non-coated or laser-exposed without coating PEEK disks. The concentration of osteocalcin secreted into the medium was also significantly higher in case of the SrHAP, SrSiP, and SrZnSiP disks than that in the case of the non-coated on day14. The calcium concentration in the PEEK disk was significantly lower in all apatite-coated disks than that in the pure PEEK disks on day 14. In qPCR, OC and ALP mRNA expression was significantly higher in the SrZnSiP disks than that in the pure PEEK disks.

**Conclusions:**

Our findings demonstrate that laser bonding of apatite—along with trace elements—on the PEEK disk surfaces might provide the material with surface property that enable better osteogenesis.

**Supplementary information:**

**Supplementary information** accompanies this paper at 10.1186/s12891-020-03716-1.

## Background

There are various materials that can be used in implants for orthopedic surgeries such as spinal fixation, joint arthroplasty, and osteosynthesis. Among them, polyether-ether-ketone or PEEK is becoming increasingly common as a material for spinal applications because PEEK has high biocompatibility, radiolucency, and elasticity properties, which are more similar to those of natural bone compared to metal materials [1]. However, because it is biologically inactive, which hampers osseointegration, its clinical use poses several risks, such as false joints, loosening, and sinking [2, 3]. For providing PEEK with the osteogenesis, various attempts, including the application of hydroxyapatite either by coating onto the surface or mixing with PEEK, have been reported [4–7]. The coating of PEEK materials with apatite is one of the most promising approaches, although its needs further research to achieve much more osseointegration and better surgical outcomes.

In recent years, we have aimed to improve the interfacial adhesion between the apatite coating and PEEK. By enhancing bone formation on the surface of the materials, such PEEK implants would facilitate osseointegration and complete healing successfully. Thus far, various attempts have also been made to add osteogenic properties to hydroxyapatite-based materials by incorporating either strontium [8] or silicate [9] ions in the crystal lattice of apatite. Recently, we reported that osteogenesis around polyethylene terephthalate artificial ligament was enhanced by silicate-substituted strontium apatite nanocoating [10]. In this study, we anticipated synergetic effects of both strontium and silicate ions and prepared silicate-substituted strontium apatite (SrSiP) disks. There are some researches reporting the effectiveness of zinc ion in osseointegration [11, 12]. Laser-assisted bonding technique [13] between polymers and ceramics improve the interfacial adhesion between the apatite coating and PEEK. However, there has been no study whether it is effective to promote osteogenesis on the PEEK disks coated apatite with strontium by using laser. We heat-fixed SrSiP on the PEEK surface by using CO_2_ laser [14]. This technique produced a new type of PEEK/apatite hybrid without any adhesives, and a bioactive and osteogenetic PEEK material might be synthesized. Then, in this study, we evaluated whether it stimulates osteogenesis on the surface of PEEK. The effects of surface modification were also studied by in vitro osteogenic cell culture experiments using rat bone marrow mesenchymal stem cells (BMSCs) [10, 15].

## Methods

### Preparation of bone marrow cells

All experimental protocols using animals were approved by the Animal Experimental Review Board of our institution before the beginning of the experiments. The animals were housed in a temperature-controlled environment at approximately 21 °C under a 12-h light/12-h dark cycle with free access to food and water. A total of 6 Fischer 344 rats weighing 160 g ± 5 g (7 weeks-old, male) were purchased from SLC Japan, Inc. (Shizuoka, Japan) for use as donors. The rats were euthanized using 4% isoflurane (Pfizer, Tokyo, Japan), which they inhaled for 5 min while placed inside a sealed container; in addition, 50 mg/kg pentobarbital (Kyoritsu Seiyaku, Tokyo, Japan) was injected into their peritoneal cavities. The deaths of the animals were confirmed based on characteristic features such as cardiac arrest, respiratory arrest, and loss of corneal reflex. BMSCs were obtained by flushing out the rat femur shafts with 10 mL culture medium consisting of minimal essential medium (Nacalai Tesque, Kyoto, Japan) containing 15% fetal bovine serum and antibiotics (100 U/mL penicillin and 100 mg/mL streptomycin; Nacalai Tesque, Kyoto, Japan). The BMSCs were incubated at 37 °C in a humidified atmosphere containing 5% CO_2_. And approximately on day 14, the primary cultured BMSCs were harvested using a trypsin-ethylenediaminetetraacetic acid (EDTA) solution (0.25% trypsin, 0.53 mM EDTA-4Na; Nacalai Tesque, Kyoto, Japan) to obtain cell suspension [10, 145]. Cell culture experiments below using this cell suspension were repeated three times (Supplementary table).

Two rats were needed to obtain the cell suspension for the single series of the experiment, therefore a total of 6 rats were sacrificed.

### Preparation of PEEK disks

PEEK disks (13 mm diameter) were prepared according to a previous study [14]. First, PEEK disks were coated with the 6% of hydroxyapatite (HAP), 6% of strontium hydroxyapatite (SrHAP), 6% of silicate-substituted strontium apatite (SrSiP), or 6% of silicate-zinc-substituted strontium apatite (SrZnSiP) and dried at room temperature. Then, the coated surfaces were irradiated by CO_2_ laser (3-Axis CO_2_ Laser Marker ML-Z9510; Keyence, Osaka, Japan). The output power was 30 W and the exposure energy was adjusted by controlling the scanning speed and the working distance between the laser output and the PEEK disks. During the laser exposure, the surface temperature at the irradiating spot was monitored in real time by using a high-speed digital pyrometer (IMPAC IGA6/23 Advanced; LumaSense Technologies Inc., CA, USA) and analyzed by a digital oscilloscope (SDS1000CML; Siglent Technologies Co. Ltd., OH, USA). After laser exposure, the PEEK disks were washed thoroughly with acetone and dried. Further, non-coated PEEK disks and laser-exposed PEEK disks without apatite coating were prepared as the negative controls. We prepared 6 disks for each group (non-coated, laser-exposed without apatite coated, HAP, SrHAP, SrSiP and SrZnSiP).

### Culture on PEEK disks

BMSCs were seeded on the prepared PEEK disks (*n* = 6 for each group), as mentioned above paragraph, at a density of 1 × 10^4^ cells/cm^2^ in 24-well culture plates (Falcon, BD Biosciences, NJ, USA) and cultured in osteogenic medium containing 10 nmol/L dexamethasone (Dex, Sigma, MO, USA), 0.28 mmol/L l-ascorbic acid phosphate magnesium salt n-hydrate (AscP, Wako Pure Chemical Industrials, Kyoto, Japan) and 10 mmol/L β-Glycerol phosphate disodium salt pentahydrate (β-GP, Sigma, MO, USA) for 14 days in 24-well plates. On day 6, the PEEK disks were transferred to new plates in order not to be reflected the spilled cells from the PEEK disks to plates.

### Scanning electron microscopy (SEM)/energy dispersive X-ray spectrometer (EDS)

Scanning electron microscopy (SEM)/energy dispersive X-ray spectrometer (EDS) observations were made after laser irradiation and after 14 days of cell culture, as mentioned above, in all 6 groups (non-coated, laser-exposed without apatite coated, HAP, SrHAP, SrSiP and SrZnSiP). SEM was performed using a low vacuum scanning electron microscope (SU3500; Hitachi, Tokyo, Japan) equipped with EDS (Octane Plus; Ametek Inc., PA. USA), with acceleration voltage of 20 kV at 60 Pa [14].

### Staining of PEEK disks

After 14 days’ cell culture on each PEEK disk as mentioned above, for alkaline phosphatase (ALP) staining [16], the PEEK disks were rinsed twice with phosphate buffered saline (PBS), and then stained with naphthol-AS-MX phosphate sodium salt (Sigma, MO, U.S.A) and fast red violet LB salt (Nacalai Tesque, Kyoto, Japan) and 0.056 mol/L 2-amino-2-methyl-1,3-propanediol buffer (AMP buffer, pH 9.9; Wako Pure Chemical Industrials, Kyoto, Japan) at room temperature for 10 min. The stain was removed by rinsing with tap water, and the samples were air-dried.

For alizarin red staining [15], as well as ALP staining, the PEEK disks were rinsed twice with PBS, and then stained with Alizarin red S (Nacalai Tesque, Kyoto, Japan) and PBS at room temperature for 10 min. Subsequently, the stained disks were rinsing with tap water and air-dried.

Furthermore, the strength of staining were compared between the PEEK disks with osteogenic cells culture group as mentioned above and the negative control groups of the PEEK disks without cells in all 6 groups (non-coated, laser-exposed without apatite coated, HAP, SrHAP, SrSiP and SrZnSiP), respectively. The PEEK disks of negative control group were also cultured in the osteogenic medium for 14 days in the same manner as that of the PEEK disks with cells.

### Biochemical analysis

The osteocalcin (OC) content of the culture medium was measured by an ELISA method [17] (DS Pharma Biomedical, Osaka, Japan) developed in a previous study [18]. Secreted OC levels were measured on days 8 and 14 and the experiments are repeated three times. The culture plates were changed 2 days before collecting medium samples for analysis (*n* = 6 for each group) to reduce the influences of the fallen cells out of the disks. On days 8 and 14, total calcium content of the culture medium was measured using the methylxylenol blue method (Calcium E-test Wako Kit; Wako Pure Chemical Industrials, Kyoto, Japan) (*n* = 6 for each group). In the past study, very high correlation between the estimated cumulative calcium reduction and the osteogenic activity in vivo (ALP activity and OC content) was repotted (*r* > 0.90) [19]. Therefore, since reduced calcium in the culture medium reflects the amount of calcium deposited, the calcium concentration in the culture medium can be used as a marker of osteogenesis in tissue-engineered bone [19].

### Gene expression analysis

The gene expression level of OC, ALP, Runt-related transcription factor-2 (Runx2), collagen type 1a1 (Col1a1), and collagen type 4a1 (Col4a1) were evaluated [19]. Total RNA was extracted from the cultured cells (RNeasy Micro Kit (50) 74004; QIAGEN, Hilden, Germany) and converted to cDNA using oligo-dT primers (oligo-dT primers (Promega, USA)) according to the manufacturer’s protocol (*n* = 3 for each group). To measure the gene expression levels, real-time quantitative PCR (ABI Step One Plus Real Time PCR System; Thermo Fisher Scientific, Waltham, MA, USA) was performed using primers as follows. The primers for the target mRNAs were OC (Rn01455285 g1; Thermo Fisher Scientific, Waltham, MA, USA), ALP (Rn00564931 m1), Runx2 (Rn01512298 m1), Col1a1 (Rn01463848 m1), Col4a1 (Rn01482925 m1), and GAPDH (Rn99999916 s1). The expression levels of each target gene were standardized with respect to glyceraldehyde-3-phosphate dehydrogenase (GAPDH) mRNA expression level.

### Statistical analysis

The results were statistically analyzed using one-way ANOVA, and post hoc multiple comparisons were performed using Dunnett test with IBM SPSS Statistics 25 (IBM, IL, USA). Statistical significance was set at *p* < 0.05. The OC and calcium concentrations of laser-exposed and with or without apatite coating PEEK groups were compared to the non-coated PEEK group. Similarly, the mRNA of laser-exposed and with or without apatite coating PEEK groups were compared to the non-coated PEEK group.

## Results

### Staining of PEEK disks

**Fig. 1:**
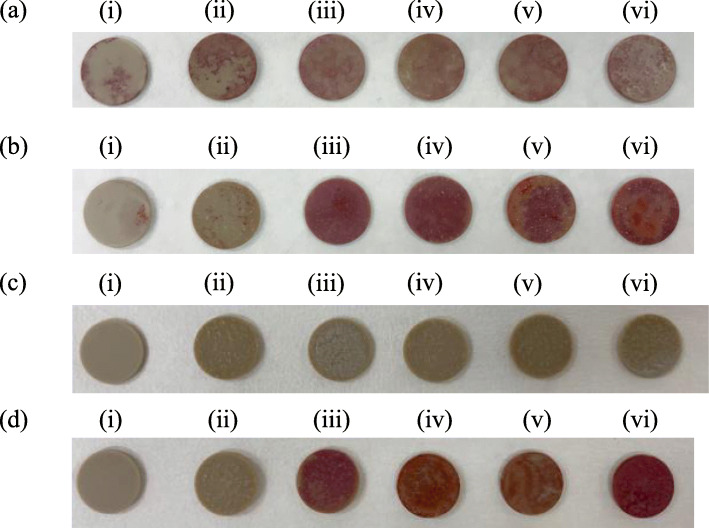
ALP staining (**a**) and Alizarin red S staining (**b**) on day 14 of cell culture, and ALP staining (**c**) and Alizarin red S staining (**d**) on day 14 without cells culture. (i) Non-coated plain PEEK disk (control); (ii) lase-exposed without apatite coating PEEK disk; (iii) 6% HAP coated PEEK disk; (iv) 6% SrHAP-coated PEEK disk; (v) 6% SrSiP-coated PEEK disk; and (vi) 6% SrZnSiP-coated PEEK disk. ALP and Alizarin red S staining were more strongly positive on the apatite-coated PEEK disks than that on non-coated or laser-exposed without coating PEEK disks (**a**, **b**). On the PEEK disks without cells culture, ALP were not stained on all disks (**c**). Alizarin red S were not stained on non-coated or laser-exposed without coating PEEK disks without cells culture, but were positive on the apatite-coated PEEK disks (**d**)

As seen in Fig. 1a-b, ALP and Alizarin red S were more strongly positive on the PEEK disks coated with apatite compared to non-coated or laser-exposed without coating PEEK disks. Furthermore, seen in Fig. 1c, disks were not stained for ALP in all 6 groups without cells. As for Alizarin red S, seen in Fig. 1d, disks were not stained on non-coated or laser-exposed without coating PEEK disks without cells culture, but were positive on the apatite-coated PEEK disks.

### SEM/EDS observation

**Fig. 2:**
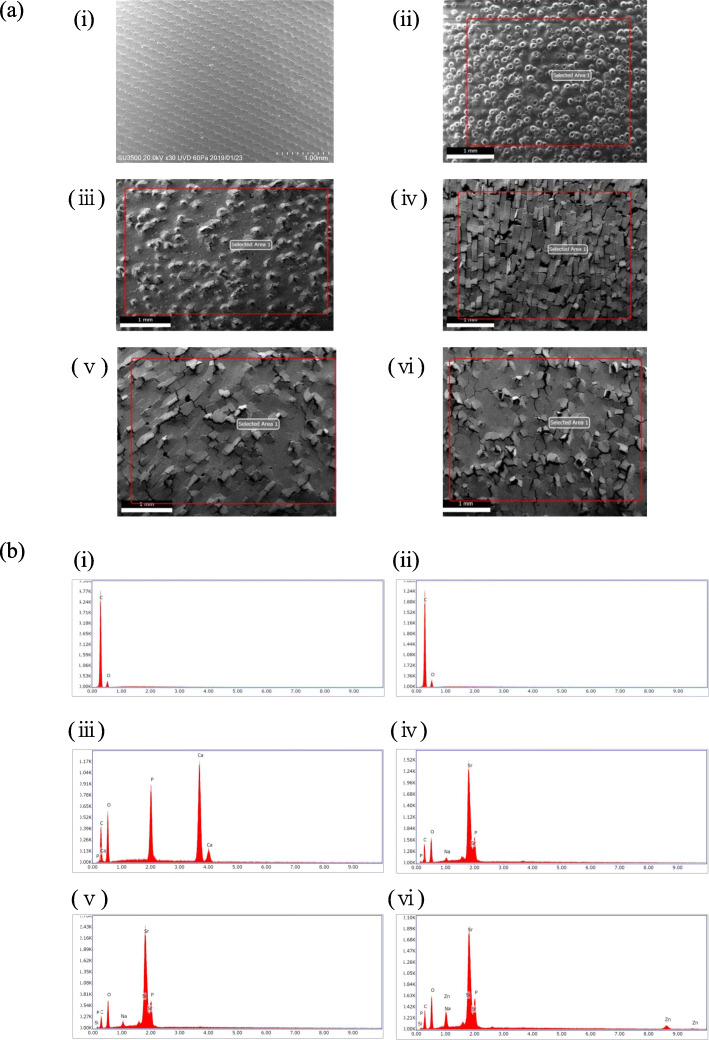
SEM/EDS analyses of PEEK surfaces after laser irradiation. **a** SEM observation before cell culture on the disks: (i) non-coated PEEK, (ii) laser-exposed without coating PEEK, (iii) HAP-coated PEEK, (iv) SrHAP-coated PEEK, (v) SrSiP-coated PEEK and (vi) SrZnSiP-coated PEEK disks. The indicated area in the figure were analyzed, and (**b**) the elemental compositions are shown in the figures. The concentration of apatite in the coating solution was 6.4 wt%

Figure 2a shows the SEM observations before cell culture of PEEK disks; only non-coated PEEK wasn’t been irradiated (Fig. 2a(i)). As shown in the laser-exposed PEEK disk images (Fig. 2a(ii)-(vi)), the coating layer presented many cracks. The elemental composition of the surface determined by EDS analysis (Fig. 2b) showed only trace amounts of carbon.

**Fig. 3:**
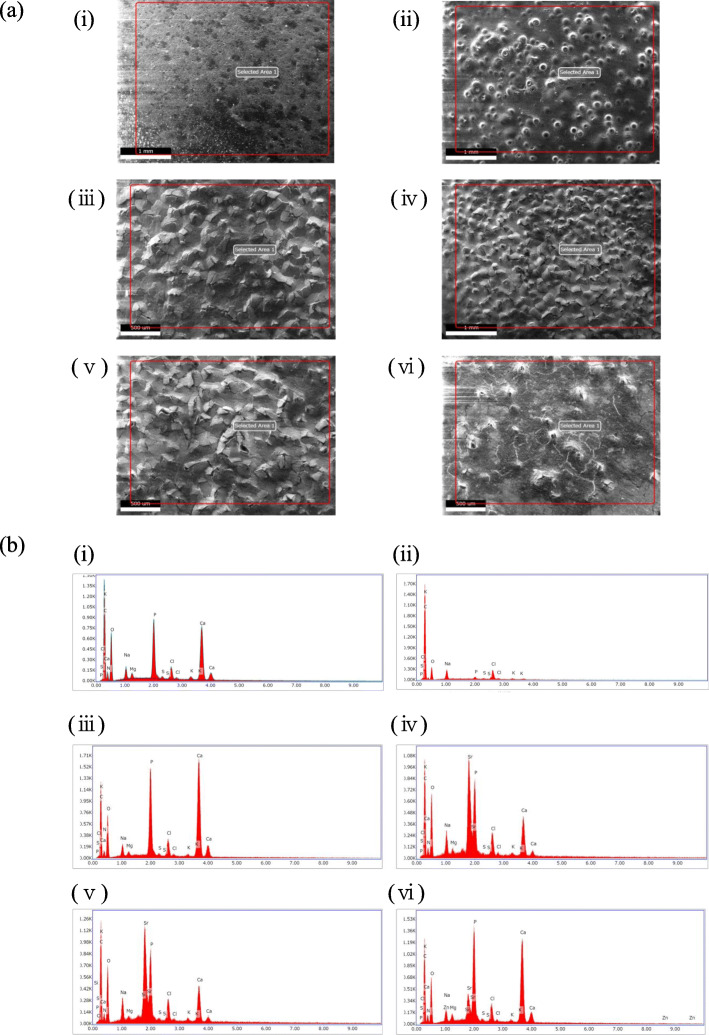
SEM/EDS analyses of PEEK surfaces after 14 days of cell culture. **a** SEM observation after cell culture on the disks: (i) non-coated PEEK, (ii) laser-exposed without coating PEEK, (iii) HAP-coated PEEK, (iv) SrHAP-coated PEEK, (v) SrSiP-coated PEEK and (vi) SrZnSiP-coated PEEK disks. The indicated area in the figure were analyzed, and (**b**) the elemental compositions are shown in the figures. The surfaces were covered with deposited calcium phosphate and extracellular matrix (ECM). Apatite underneath the deposits was slight detected by SEM (iii-vi), but clearly more detected than non-coated and laser-exposed without coating PEEK by EDS

Figure 3a shows the SEM observations of the surface of the PEEK disks after 14 days of cell culture. The disk surfaces were covered with a thick layer of deposits produced during cell proliferation. EDS analysis (Fig. 3b(iii)-(vi)) confirmed extensive deposition of CaP via the bio-mineralization process, especially in HAP and SrZnSiP-coated PEEK disks.

### Biochemical analysis

**Fig. 4:**
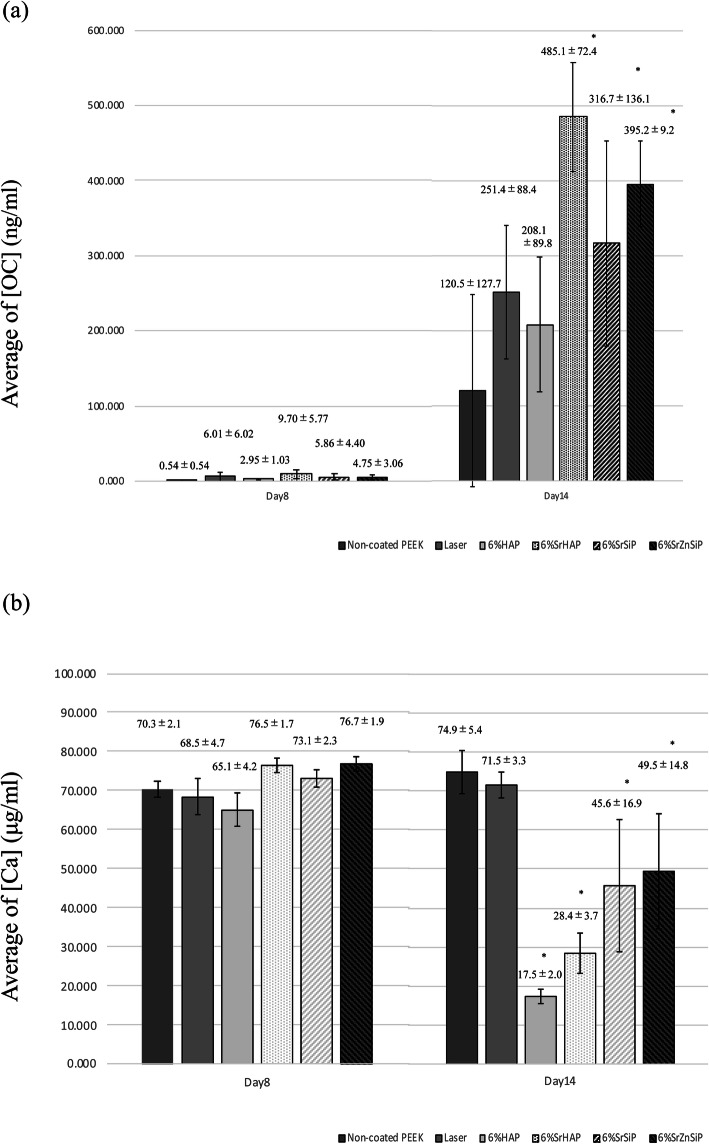
OC and calcium concentration in culture medium (*n* = 6 for each group). **a** OC concentration in each group on day 8 and day 14. The comparison with non-coated PEEK (control group). There were no significant differences among the groups on day 8, but on day 14, the concentration of OC increased in all groups. Compared to the control group (non-coated group), the OC concentration was significantly higher in the SrHAP, SrSiP, and SrZnSiP groups on day 14. Data are shown as the mean ± SD. Asterisk indicates *p* < 0.05 vs. non-coated PEEK group. **b** Calcium concentration in each group on day 8 and day 14. The comparison with non-coated PEEK (control group). The concentration of calcium was determined on day 8 and on day 14. There were no significant differences among groups on the day 8, but the calcium concentration in the apatite-coated groups tended to decrease on day 14. Compared to the control group (non-coated group), the calcium concentration was significantly lower in the HAP, SrHAP, SrSiP, and SrZnSiP groups on the day 14. Data are shown as the mean ± SD. Asterisk indicates *p* < 0.05 vs. non-coated PEEK group

The concentration of OC in the culture medium was not significantly different among the groups on day 8, but on day 14, the concentration increased in all groups (Fig. 4a). Furthermore, compared to the non-coated group, the OC concentration was significantly higher in the SrHAP, SrSiP, and SrZnSiP groups on day 14 (Fig. 4a).

Similarly, the calcium concentration in the culture medium was not significantly different among the groups on day 8. On day 14, the concentration of calcium in the apatite-coated groups tended to decrease in contrast with the non-coated and laser-exposed without apatite coating groups (Fig. 4b). The concentration of calcium on day 14 was significantly lower in the HAP, SrHAP, SrSiP, and SrZnSiP groups than in the non-coated group (Fig. 4b). These results indicate enhanced consumption of calcium ions by osteoblast cells cultured on the apatite-coated PEEK disks.

### Gene expression analysis

**Fig. 5:**
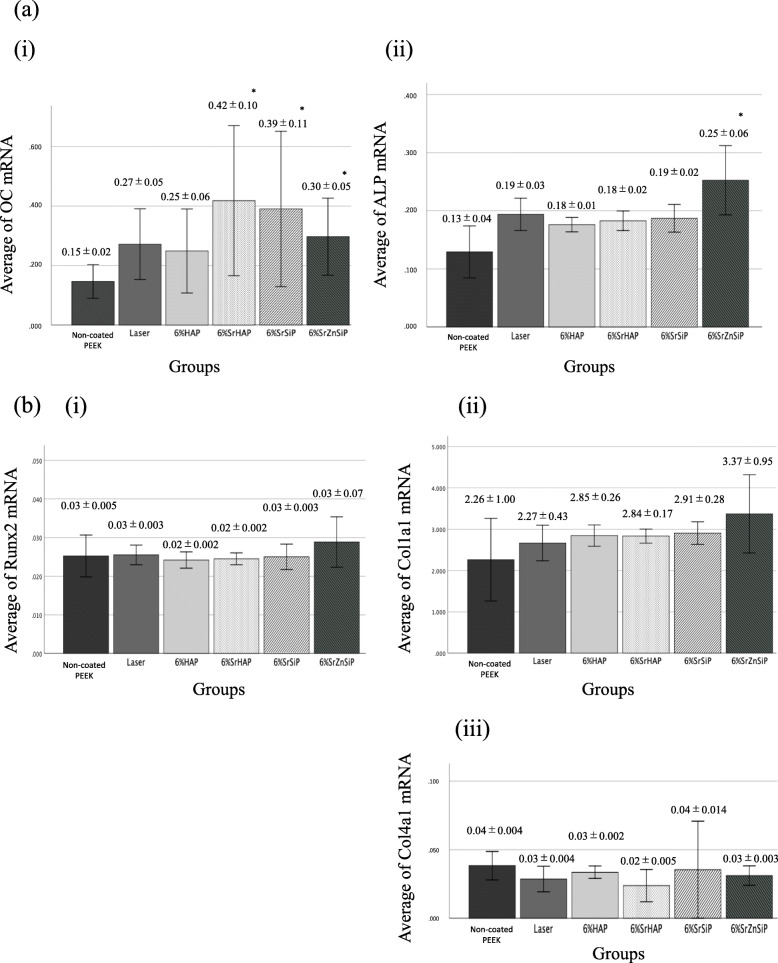
mRNA expression in the culture medium on day 14 (*n* = 3 for each group). **a** Expression of (i) OC, (ii) ALP, and (**b**) expression of (i) Runx2, (ii) Col1a1, (iii) Col4a1. The expression of OC mRNA in SrHAP, SrSiP and SrZnSiP group and the expression of ALP mRNA in the SrZnSiP group were significantly higher than that in non-coated PEEK group. However, there was no significant difference with respect to Runx2, Col1a1 and Col4a1 mRNA expressions. Data are shown as the mean ± SD. Asterisk indicates *p* < 0.05

We performed osteogenic gene expression analysis on day 14 and found that the OC and ALP gene expression in the SrZnSiP group was significantly higher than in the non-coated PEEK group (Fig. 5a). However, the level of Runx2, Col1a1, and Col4a1 expression was the same in all groups compared to the non-coated PEEK group (Fig. 5b).

## Discussion

PEEK was developed in the UK in 1987, and Brantigan et al. [20] reported carbon-reinforced PEEK as a material for lumbar interbody cages in 1991. PEEK has been widely used because of its high biocompatibility, radiolucency, and elasticity properties, which are more similar to those of natural bone compared to metal materials. However, some issues have been pointed out in terms of osseointegration. Therefore, it is important to develop a technique to provide PEEK with suitable surface properties to enhance osseointegration. Collectively, the results of this study revealed that laser bonding of apatite coating on PEEK surface might provide osseointegration to PEEK.

Some PEEK surface modifications have been reported, and these include physical treatment, chemical treatment, and surface coating. Briem et al. treated the PEEK surface with plasma and reported differentiation of primary fibroblasts and osteoblasts on the plasma-treated PEEK [21]. After this study, other types of plasma have been reported, such as oxygen plasma [22, 23] and oxygen/argon plasma [24]. Khoury et al. employed the accelerated neutral atom beam technique [25, 26], which successfully enhanced the surface bioactivity of PEEK without modification of the surface chemistry. Furthermore, laser-assisted biomimetic process [27] and vacuum plasma spray coating [7] were reported.

Various materials have been deposited on the surface of PEEK to enhance osseointegration; these include apatite (including HAP), titanium (Ti), and gold, among others. The most commonly material used as a coating for PEEK is HAP, and many studies have consistently shown that HAP typically exhibits excellent biocompatibility, bioactivity, and osteoconduction in vivo [28, 29].

In our study with the laser bonding technique [14], a strong bonding between apatite and PEEK surface was achieved without any adhesives and produced a new type of PEEK/apatite hybrid with bioactive and osteogenetic PEEK material. Furthermore, the trace elements’ ions in the apatite layer, i.e., strontium, silicon, and zinc, might promote osteogenesis around PEEK disks probably as a result of the bioactivity of these apatites. The results of ALP staining (with cells vs without cells) indicate that apatite coating might be able to promote bone formation on the PEEK disks. On the other hand, the positive staining of Alizarin red S of the control without cells might be attributed by the calcium ion for HAP or strontium ion for SrHAP, SrSiP and SrZnSiP, respectively, contained in the coating apatite itself. Indeed, the manufacture’s instruction for Alizarin red S staining indicates that metal elements other than calcium, such as strontium, cause the positive staining. Strontium enhances the secretion of osteoprotegerin and inhibits the differentiation of osteoclasts [30]. Silicon not only stimulates bone formation in osteoblasts, but also has inhibitory effects on osteoclasts [31]. Furthermore, silicon is known to enhance the bioactivity and osteogenic properties of materials [32, 33]. Zinc enhances the anti-osteoclastic effect of phytoestrogens and may limit aspects of their anabolic action on bone matrix formation [34]. The results of this study indicate that each apatite including strontium, zinc, and silicon might be able to promote bone formation.

There are several limitations to this study. First, biomechanical evaluation of the laser bonding has not been performed. In the future, we will evaluate the coating biomechanical properties and further test them using in vivo experimental models in accordance with the relevant guidelines. Second, the amount of apatite on the PEEK disk has not been measured. We should apply the same coating technique consistently and to accurately evaluate the quality of the coating. The lack of uniformity in the laser coating would cause the data variations. From this study, we indicated SrZnSiP might be the best apatite to promote bone formation. Therefore, in the future, we’re going to study how to coat uniform SrZnSiP laser coating. Third, there is no evidence for elution of trace elements’ ion from the apatite layer. We should investigate it next. Finally, it is necessary to investigate further whether there is any related adverse event.

## Conclusion

This study suggests that strontium apatite bonded by using the CO_2_ laser on PEEK surface might be a technique providing it with surface properties for better osteogenesis. Among them, SrZnSiP would be the best apatite to promote bone formation. As a result, there is a possibility that this technique might lead to better outcomes in surgery.

## Supplementary information

**Additional file 1.**

## Data Availability

The datasets used during the present study are available from the corresponding author on reasonable request.

## Abbreviations

ALP: Alkaline phosphatase
BMSCs: Bone marrow mesenchymal cells
Col1a1: Collagen type 1a1
Col4a1: Collagen type 4a1
ECM: Extracellular matrix
EDS: Energy dispersive X-ray spectrometer
EDTA: Ethylenediaminetetraacetic acid
GAPDH: Glyceraldehyde-3-phosphate dehydrogenase
HAP: Hydroxyapatite
OC: Osteocalcin
PBS: Phosphate buffered saline
PEEK: Polyether-ether-ketone
qPCR: Quantitative polymerase chain reaction
Runx2: Runt-related transcription factor 2
SD: Standard deviation
SEM: Scanning electron microscopy
SrHAP: Strontium hydroxyapatite
SrSiP: Silicate-substituted strontium
SrZnSiP: Silicate-zinc-substituted strontium apatite
Ti: Titanium
