# Supplementary material for: In vitro osteogenesis of rat bone marrow mesenchymal cells on PEEK disks with heat-fixed apatite by CO2 laser bonding
Source: BMC Musculoskelet Disord. 2020 Oct 19;21:692. doi: 10.1186/s12891-020-03716-1 (PMC7574580; doi:10.1186/s12891-020-03716-1)
Supplement: Supplementary file 1 — Additional file 1. [file 12891_2020_3716_MOESM1_ESM.docx]

|  | SEM/ EDS Observation | Staining | | Biochemical Analysis | | Gene Expression Analysis |
| --- | --- | --- | --- | --- | --- | --- |
|  |  | ALP | Alizarin Red | OC | Ca | mRNA |
| Day | 0 and 14 | 14 | 14 | 8 and 14 | 8 and 14 | 14 |
| Technical Replication | 1 | 1 | 1 | 6 | 6 | 3 |
| Biological Replication | 1 | 3 | 3 | 3 | 3 | 3 |
